# Interaction of smoking and occupational noise exposure on hearing loss: a cross-sectional study

**DOI:** 10.1186/1471-2458-7-137

**Published:** 2007-07-03

**Authors:** Gholamreza Pouryaghoub, Ramin Mehrdad, Saber Mohammadi

**Affiliations:** 1 Department of Occupational Medicine, Tehran University of Medical Sciences, Tehran, Iran

## Abstract

**Background:**

Noise is the most common hazardous agent at workplaces. Noise induced hearing loss (NIHL) has been known since the industrial revolution. Although NIHL is permanent, irreversible and frequent, it is preventable. The economic costs of NIHL have been estimated to be about billions of dollars. Besides, cigarette smoking is a common habit worldwide, and according to some recent studies smoking and noise may act in common causal pathways for hearing loss.

**Methods:**

A cross-sectional study was designed to study the effect of smoking on NIHL in 206 male smoker workers and 206 male non-smoker workers in a large food-producing factory, in which workers were exposed to noise levels exceeding 85dBA. To determine noise exposure level, we used sound level measurements reported by industrial hygienists.

A qualified audiologist assessed hearing acuity by using standardized audiometric procedures assuring at least 14 h of noise avoidance.

**Results:**

We observed that the percentage of workers with hearing threshold differences of greater than or equal to 30 dB between 4000 Hz and 1000 Hz in both ears were 49.5% and 11.2% in smoker and non smoker groups, respectively (Odds ratio = 7.8, 95% CI = 4.7 – 13), and the percentage of workers with a hearing threshold of greater than 25dB at 4000 Hz in the better ear were 63.6% and 18.4% in smoker and non smoker groups, respectively. This difference was statistically significant after adjustment for age and exposure duration.

**Conclusion:**

It can be concluded that smoking can accelerate noise induced hearing loss, but more research is needed to understand the underlying mechanisms. Accurate follow up of smoker workers who are exposed to noise levels exceeding 85 dBA is suggested. Smokers should periodically attend educational courses on "smoking cessation", especially in noisy workplaces.

## Background

Noise is the most pervasive hazardous agent at workplaces [1,2]. Approximately 30 million American workers are exposed to hazardous noise on their jobs [3,4], and it is estimated that approximately 600 million workers are exposed to occupational noise worldwide [5].

Generally, NIHL (Noise- Induced Hearing loss) is a sensorineural hearing deficit which begins at higher frequencies (3,000 to 6,000 Hz) and develops gradually as a result of chronic exposure to excessive sound levels [6]. The mechanism by which the sensory cells are lost is probably a combination of physical and metabolic stress. There are large number of studies that suggest the production of reactive oxygen species in response to noise overexposure plays a role in this process [7,8]. Early NIHL at the 3000–4000 Hz region [9] of the audiogram correlates with hair cell degeneration of the organ of corti [10] located near the base of the cochlea. Individual susceptibility to noise-induced hearing loss varies greatly, but the reason that some persons are more resistant to it while others are more susceptible is not well understood [11].

According to NIOSH's (National Institute of Occupational Safety & Health) estimate, 14% of the working population in the USA is employed in environments where the A-weighted sound exposure level exceeds 90 decibels. In some manufacturing plants such as those producing textile mill products, lumber and wood products and food and kindred products this ratio exceeds 25% [12,13]. Likewise NIHL can affect the quality of life in workers and causes problems such as social isolation, depression and an increased risk of accidents [14,15]. Although NIHL is permanent, irreversible and frequent, it is preventable [16]. Besides, the economic costs of occupational hearing loss have been estimated to be about billions of dollars [17-19]. On the other hand, cigarette smoking is a common habit worldwide. In general, tobacco is consumed by approximately 1.3 billion of the world population [20]. Tobacco may also affect cochlear blood supply because it causes peripheral vascular changes, such as increased blood viscosity [21], and reduced oxygen availability. These effects were identified as the etiology of cochlear lesions in laboratory animals and humans [22,23]. Therefore, smoking and noise may act in common causal pathways for hearing loss, through the reduction of cochlear blood supply. Although studies have reported a positive association between smoking and hearing loss [24-28], the combined effects involving smoking and noise have rarely been assessed [29,30]. The main object of this study is to examine the hypothesis that smoking and noise jointly affect hearing, which could be of particular relevance in noise control programs for workers.

## Methods

This cross-sectional study was conducted in a large food-producing factory situated in the capital city of Iran. There were no other known occupational hazards affecting hearing acuity, but noise. Noisy work-places were selected based on industrial hygienists' reports on sound level measurements; these measurements were performed annually, and saved as "noise monitoring records". In this study, the last 3 records were used and all 6 parts of the factory in which the noise levels exceeded 85 dBA in all 3 records, were selected as noisy workplaces. At different stations of these 6 parts the lowest and highest noise levels were 87dB and 94dB, respectively. Almost all non-noisy parts were offices. The turnover of workers among noisy parts was so fast that it was impossible to estimate the exact level of exposure of each worker, but the turnover of worker between noisy and non-noisy parts was slow and exposure duration could be calculated almost exactly. Our study population were all the male smoker workers of these noisy parts (n = 253). Female workers were not enrolled in this study because of the low noise exposure in this group (almost all women were office workers). After filling in the questionnaire, all workers with a history of ototoxic drug use, diabetes mellitus, hyperlipidemia, hypothyroidism, severe or frequent ear infection, ear surgery, exposure to non-occupational noise (such as amplified music, participation in war, hunting, etc.), noise exposure in previous job/jobs, unilateral or conductive hearing loss or any kind of hearing loss with a known etiology except noise exposure were excluded (n = 47). 206 male smoker workers who worked in noisy workplaces and participated in periodic medical examinations became the objective population. All the subjects participated voluntarily in this study and an inform consent was filled in by each (written consent in Persian was obtained).

Smoking was ascertained based on the following questions: 'Have you ever smoked?' and 'Do you smoke?' Duration of smoking, working in noisy workplaces and age were recorded in years. A qualified audiologist assessed hearing acuity using standardized audiometric procedures assuring at least 14 hours of noise avoidance. The exams were done in an isolated acoustic room meeting the ANSI S3.1–1991 standards with a diagnostic audiometer (Model AD 229e, interacoustic Denmark Co. Ltd). The pure-tone hearing thresholds at 500, 1000, 2000, 3000, 4000, and 6000 Hz were measured for air and bone conduction, in both ears.

The hearing threshold differences of greater than or equal to 30 dB between 4000 Hz and 1000 Hz in both ears, hearing threshold level of greater than 25dB at 4000 Hz in the better ear, and hearing threshold at 4000 Hz in the better ear were used as the indicators of hearing loss. After the initial review of the results of our study we decided to complete our study by choosing non-smoker workers as a reference group. There were 508 male non-smoker workers in these noisy parts. So 206 non-smoker male workers, who worked in the same parts were randomly selected as the reference group (42 workers were excluded by applying the exclusion criteria). Inclusion and exclusion criteria for this group were the same as the smokers group. Data collection method was also the same. Thus the only difference between the two groups was cigarette smoking. This study was approved by the Ethics Committee of Tehran University of Medical Sciences. Data were analyzed using SPSS version 11.5. The following statistical tests were applied in analysis of the data presented in this article. The two-sample t test was applied for testing differences between the study groups for quantitative parameters. Pearson χ^2 ^test was applied to examine differences between the study groups for categorical parameters. Association of hearing loss with smoking habit was examined by binary logistic regression. The modeling strategy followed Hosmer and Lemeshow [31]. All variables (age, exposure duration and smoking as categorical variables) were included in a logistic regression model. Based on Wald-test all variables had statistically significant effect on hearing loss (hearing threshold of greater than 25 dB at 4000 Hz in the better ear) as a dependent variable. None of variables removed, and all of them are included in the final model. We used Hosmer and Lemeshow's goodness of fit test to examine the adequacy of model. All tests applied were two-tailed, and a P value of 0.05 was considered statistically significant.

## Results

The percentage of workers with hearing threshold differences of greater than or equal to 30 dB between 4000 Hz and 1000 Hz in both ears were 49.5% in smokers and 11.2 % in non-smokers (odds ratio = 7.8, 95% CI = 4.7 – 13), and the percentage of hearing threshold level of greater than 25dB at 4000 Hz in the better ear were 63.6% and 18.4% in smoker and non-smoker groups, respectively (odds ratio = 7.7, 95% CI = 4.9 – 12.1). Descriptive statistics of all 412 workers are shown in Table 1.

**Table 1:** Descriptive statistics of all workers (n = 412)

	**Smokers (n = 206)**		**Non-smokers (n = 206)**		**P value**
		
	**Mean (SD)**	**Range**	**Mean(SD)**	**Range**	
**Age (year)**	42.7(6.2)	24–62	41.4(7.2)	26–67	0.04
**Noise exposure duration (year)**	18.6(6.6)	2–30	17.1(6.9)	2–32	0.02
Hearing threshold at 4000 Hz (dB)*	34.1(16.4)	0–80	16.4(12.3)	0–65	< 0.001
**Smoking (Pack-Year)**	13.6(10.6)	0.3–78	----	----	----

* Better ear

As shown in Table 1, means of age and noise exposure duration in non-smokers were less than smokers (1.3 and 1.5 year respectively). Means of hearing threshold at 4000 Hz, age and noise exposure duration were compared between smokers and non-smokers using t-test (Table 1).

Age-related hearing loss (presbycusis) is one of the most common causes of high frequency hearing loss and its effects begin around the age of 40. 57.3% of non-smokers and 68.4% of smokers were older than 40, and this difference was statistically significant. To adjust the effect of age, we divided all workers into 2 groups; aged 40 or less (n = 153) and older than 40 (n = 259) and then each group was divided into 2 subgroups; smokers and non-smokers. Then we compared the hearing threshold difference between 4000 Hz and 1000 Hz of greater than or equal to 30 dB in both ears, hearing threshold of greater than 25dB at 4000 Hz in the better ear by chi square and means of hearing threshold at 4000 Hz by t-test between the two subgroups of smoker and non-smoker workers. This was done separately for the two age groups of 40 or less and older than 40 (Table 2).

**Table 2:** Comparison of hearing threshold between smoker and non-smoker workers in two separate age groups; aged 40 or less and older than 40

	**Workers aged 40 or less**				**Workers older than 40**			
	
	**Smokers (n = 65)**	**Non smokers (n = 88)**	**P value**	**Odds (%95CI)**	**Smokers (n = 141)**	**Non smokers (n = 118)**	**P value**	**Odds (%95CI)**
Diff. 4k-1k^1 ^n (%)	17 (26.2)	3 (3.4)	< 0.001	10(2.8–36)	85 (60.3)	20 (16.9)	< 0.001	7.4(4.1–13.4)
> 25 dB at 4k^2 ^n (%)	23 (35.4)	6 (6.8)	< 0.001	7.5(2.8–9.8)	108 (76.6)	32 (27.1)	< 0.001	8.8(5–15.4)
4k mean^3 ^(SD)	24.5 (14.9)	12.2 (10.1)	< 0.001	----	38.5 (15.2)	19.5 (12.9)	< 0.001	----

1- Hearing threshold difference between 4000 Hz and 1000 Hz of greater than or equal to 30 dB in both ears

2- Hearing threshold of greater than 25dB at 4000 Hz in the better ear

3- Mean of hearing threshold at 4000 Hz (dB) in the better ear

Association of hearing loss with smoking was examined by logistic regression. The confounders adjusted in model were age and exposure duration as categorical variables. Binary logistic regression was used to examine the dose-response relationship between smoking and hearing threshold of greater than 25 dB at 4000 Hz in the better ear controlled for age and noise exposure duration. Based on Hosmer and Lemeshow's goodness of fit test, the model has adequate fit. According to the smoking history workers were divided into 3 groups of non-smokers, 10 pack-years or less smoking and more than 10 pack-years smoking. Based on noise exposure duration they were divided into 3 groups of up to 10 years, 11–20 years and more than 20 years exposure. Age categories were defined as workers aged 40 or less and workers aged more than 40. The results of logistic regression are presented in Table 3.

**Table 3:** Odds Ratio (OR) and 95% Confidence Interval (CI) for hearing threshold of greater than 25 dB at 4000 Hz in the better ear with logistic regression

**variable**	**B**	**S.E.**	**Sig.**	**OR (95% CI)**
**Age**				
≤ 40 (n = 153)				1.0
> 40 (n = 259)	0.982	0.312	0.002	2.7 (1.4–4.9)
**Exposure duration**				
≤ 10 (n = 70)				1.0
11–20 (n = 177)	1.139	0.462	.014	3.1 (1.3–7.7)
≥ 21 (n = 165)	1.699	0.474	< 0.001	5.5(2.1–13.8)
**Smoking (pack/year)**				
Non smoker(n = 206)				1.0
≤ 10 (n = 105)	1.401	0.297	< 0.001	4 (2.2–7.2)
> 10 (n = 101)	3.041	0.346	< 0.001	21 (10.6–41.2)
**constant**	-3.400	0.493	< 0.001	0.033

R square = 0.47

As shown in Table 3, the associations of hearing loss with smoking, age, and noise exposure duration were significant in the logistic regression. And by increasing pack-year odds of hearing threshold of greater than 25 dB at 4000 Hz was increased.

## Discussion

In this study, the percentage of workers with difference between hearing threshold levels at 4000 Hz and 1000 Hz of greater than or equal to 30 dB in both ears was 49.5% in smokers, which is higher than other similar studies [24-27]. It could be due to cigarette smoking or different noise exposure intensity and duration. We selected both groups from the same workplace with identical working conditions, so that the intensity of noise exposure (noise level exposure) in these groups was almost the same.

Also in workers exposed to noise levels exceeding 85 dBA, the risk of high frequency hearing loss is increased in a statistically significant manner in relation to increasing pack-years of smoking. According to literature, age-related hearing loss (presbycusis) is one of the most common causes of high frequency hearing loss and its effects begin around the age of 40 [32,33]. The differences of means of age and noise exposure duration between smokers and non-smokers were 1.3 and 1.5 years respectively which were statistically significant but this couldn't explain the observed difference in the three hearing loss indicators between the groups and the effect of smoking on hearing loss remains after adjustment for age and noise exposure duration.

## Conclusion

It can be concluded that smoking may accelerate noise induced hearing loss. The cross- sectional design of this study does not permit causal inference from the observed associations. But the observed relationships give valuable evidence for further researches. If prospective studies confirm this idea, smoking cessation or modification of smoking habits may delay the appearance of hearing loss. Therefore it is recommended that in order to detect hearing loss at its early stages, smoker workers exposed to noise levels exceeding 85 dBA, should be followed carefully. It is also recommended that they should attend educational courses on smoking cessation periodically, especially in noisy workplaces

Evidences have accumulated in recent years on the adverse effects of smoking on hearing among the working population, such as studies conducted by Nakanishi N, et al in Ozaka university, Japan [24]; Barone JA, et al, in department of preventive medicine, California university, USA [25]; Hong OS, Kim MJ, in an airline industry, Korea [26]; Virokannas H, Anttonen H, in Oulo university, Finland [27]; Mizoue T, et al, in Kyushu university, Japan [34]; Palmer KT, et al, in Southampton university, England [28]; Noorhassim I, Rampal KG, national university of Malaysia [35]; and Ferrite S, Santanna V, Bahia university, Brazil [36]. A few of studies haven't found any association between smoking and hearing loss, such as Karlsmose B, et al, in Denmark [37].

A possible explanation for the underlying pathogenic mechanism may be the well-known vascular changes and the consequent cochlear hypoxia related to both smoking and long-term intense noise exposure [21,22,38-40].

Ultimately it can be mentioned that there are few studies about the combined effect of occupational exposure to noise and smoking on hearing loss. But the results of most of them (including our study) demonstrate that smoking can accelerate hearing loss with various degrees. More research is needed to understand the underlying mechanisms.

## List of abbreviations

NIHL: noise induced hearing loss

## Competing interests

The author(s) declare that they have no competing interests.

## Authors' contributions

Dr. Pouryaghoub has worked as a designer and leader of study, Dr. Mehrdad has worked specially in analyzing data, and Dr. Mohammadi has worked specially in gathering data as his residential thesis. All authors have been involved in drafting the manuscript.

## Pre-publication history

The pre-publication history for this paper can be accessed here:


